# Assessing Access Control Risk for mHealth: A Delphi Study to Categorize Security of Health Data and Provide Risk Assessment for Mobile Apps

**DOI:** 10.1155/2020/5601068

**Published:** 2020-01-16

**Authors:** Pedro Moura, Paulo Fazendeiro, Pedro R. M. Inácio, Pedro Vieira-Marques, Ana Ferreira

**Affiliations:** ^1^CINTESIS—Center for Health Technologies and Services Research, Faculty of Medicine, University of Porto, Porto, Portugal; ^2^Department of Computer Science, Universidade da Beira Interior and Instituto de Telecomunicações, Covilhã, Portugal

## Abstract

**Background:**

Smartphones can tackle healthcare stakeholders' diverse needs. Nonetheless, the risk of data disclosure/breach can be higher when using such devices, due to the lack of adequate security and the fact that a medical record has a significant higher financial value when compared with other records. Means to assess those risks are required for every mHealth application interaction, dependent and independent of its goals/content.

**Objective:**

To present a risk assessment feature integration into the SoTRAACE (Socio-Technical Risk-Adaptable Access Control) model, as well as the operationalization of the related mobile health decision policies.

**Methods:**

Since there is still a lack of a definition for health data security categorization, a Delphi study with security experts was performed for this purpose, to reflect the knowledge of security experts and to be closer to real-life situations and their associated risks.

**Results:**

The Delphi study allowed a consensus to be reached on eleven risk factors of information security related to mobile applications that can easily be adapted into the described SoTRAACE prototype. Within those risk factors, the most significant five, as assessed by the experts, and in descending order of risk level, are as follows: (1) security in the communication (e.g., used security protocols), (2) behavioural differences (e.g., different or outlier patterns of behaviour detected for a user), (3) type of wireless connection and respective encryption, (4) resource sensitivity, and (5) device threat level (e.g., known vulnerabilities associated to a device or its operating system).

**Conclusions:**

Building adaptable, risk-aware resilient access control models into the most generalized technology used nowadays (e.g., smartphones) is crucial to fulfil both the goals of users as well as security and privacy requirements for healthcare data.

## 1. Introduction

Health information systems can empower the performance and maintenance of health services, but the processing and storage of highly sensitive data raises serious concerns regarding privacy and safety of patients [1]. The healthcare industry is a prime target for medical information theft due to the systematic unpreparedness in dealing with cyber threats menacing vital data [2]. There is the need to increase the awareness and understanding that, in healthcare, the risk associated with patient data is not just about such data, but about patient care delivery, and potentially, even about the mental and physical health of the patient [3].

But risk, as the by-product of the likelihood of a vulnerability being exploited by a threat and the negative impact this can cause [4], is very difficult to calculate and maintain, especially in such a heterogeneous and high turnover environment. The risks can increase considerably when personal health-related data can be collected, processed, and stored by many types of different devices (e.g., smartphones, smartwatches, or other IoT sensors) and associated vulnerabilities, anytime and anywhere [5]. This situation is bound to be more and more frequent regarding not only the pressure put by the constant increase of aged population worldwide in need of health-related ambient assisted living products [6] but also the empowerment that current legislation and regulation on personal data protection offers individuals [7, 8].

In the healthcare domain, smartphones can bring many advantages to tackle diverse needs of stakeholders. Health professionals can use smartphones to access and manage patient records, to view exam results, to share and ask for second-opinion diagnosis, and to prescribe medications [9]. On the other hand, patients can use smartphones to manage, update, and control access to their medical records, monitor their health statistics, and view their prescriptions [10].

Regarding mHealth applications (Apps), i.e., software applications used on a mobile device for medical or other health-related purposes, the risk of disclosure and breach can be higher as no adequate security measures are yet available or these are not properly used for these devices [11–14], and a medical record has a significant higher financial value compared with, for instance, credit card data [15]. But, even if proper safeguards were available, they would still be difficult to verify and control in the hands of different millions of users around the world [16]. There is some work in the literature that focuses on using mobile Apps to assess risks (a) while managing a disease [17–20] and much less to (b) detect source threats to the quality and integrity of medical data of patients that circulate in mobile applications [3, 13]. However, this second type of risk assessment targets only part of the problem. There are currently no standard means to assess risk in every interaction between a user (e.g., a patient) and the mHealth App, both dependently and independently of the goal and content of the App.

Access control (often encompassing identification, authentication, authorization, and accountability) is the first and one of the most crucial interactions between users and mobile devices [21]. When a user requests data from an App, authorization is being constantly checked and so should risk assessment be constantly verified to adapt to the changes of the ubiquitous characteristics of mobile devices and their location. To do this, risk assessment must comprise technical, contextual, environmental, and user's profiling data to identify, at each access request of the user, what is the probability of a negative impact to occur when making that request available, within the identified conditions, at that specific moment.

Connected to this necessity, to the best of our knowledge, there are only two previous works tackling the need to include contextual elements in the risk assessment of mHealth Apps: a previous work on risk-adaptable access control [22] and a proposal of a risk framework to support clinical use of medical Apps [3]. The former is an access control model (SoTRAACE, Socio-Technical Risk-Adaptable Access Control) that integrates the described needed features, but it has only been presented at a theoretical level [22]. The latter strives to include only external elements directly related to the App in the risk assessment, such as inadequate training of the users or the usage factor of the App [3]. Nonetheless, it does not include elements such as network connection type (e.g., public Wi-Fi or protected ISP), version of operating system (OS) used, or if the user has already made that same request and, if so, what was the associated risk at that time (e.g., user's risk profiling). More work has been done since to developing a SoTRAACE prototype into a mHealth App and include a hybrid risk assessment feature.

The aim of this paper is to present a risk assessment feature integration into the SoTRAACE model, as well as the operationalization of the related mobile health decision policies. A Delphi study with security experts was performed and is presented to integrate the categorization of the data regarding the impact of security and privacy loss into the prototype, to reflect security experts' knowledge and to be closer to real-life situations and their associated risks. A simple prototype is discussed and validated on patient access control scenarios, using a fictitious mHealth application.

The next section presents background work and Section 3 describes the methods used to develop risk assessment into the SoTRAACE prototype, while Section 4 presents the results from the Delphi study as well as the implemented prototype with risk assessment in patient's access scenarios.  Section 5 discusses obtained results that go beyond the state of the art, together with the work limitations, while Section 6 concludes the paper.

## 2. Related Work

### 2.1. SoTRAACE and Access Control

The role-based access control model [23] is widely used to manage healthcare-related access control; however, its basic features are inflexible because the access control policy is hard-coded and preset into the decision logic or database restrictions. Moreover, those solutions often assume uniformity of people's devices, environments, and situational and technical conditions, which do not agree with the new mobile paradigm of anytime/everywhere, from different mobile devices and Internet wireless connections. Therefore, more flexible, adaptive, and dynamic access control models are required; some of which are already available in the literature and try to deal with this issue by including characteristics to cope with specific objectives. Attribute-based access control (ABAC) [24] is more flexible than RBAC because it uses the attributes of subjects and objects (instead of roles), together with environmental attributes, to make access decisions. Situation-based access control (SitBAC) [25] defines a situation as an abstract condition composed of user's contexts and related object contexts where patients' data access is permitted or denied, while location-based access control models use geographic information system (GIS) as a support to make the best evaluation regarding location and related parameters [26]. With the growing popularity of social network system models, relationship-based access control (RelBAC) [27] can be used to track interpersonal relationships between users and the expression of access control policies in terms of those relationships. This concept in healthcare can be reflected with the provision of a closer relationship between patients, health professionals, and patients' family. For healthcare emergency/unanticipated situations or authentication or policy errors, BTG-RBAC [28] can be used to break or override access controls in a controlled manner. Finally, there are a few models that try to adapt access control decisions according to the situation and context at the moment of request. Risk-adaptable access control (RAdAC) [29] introduces the idea of balancing security risk against operational need. This is made with the belief that the operational benefits of sharing the information outweigh the potential security risk of sharing it. Security policy grants or denies can be reversed according to the operational need and security risk at the moment of the requested access. However, neither this model nor the more complete version described in [30] includes all factors together, e.g., social and behavioural factors, trust levels, granularity of the objects, devices with different OSs, location, or even the BTG (break the glass) component, to aid making the most accurate and adaptable access control decisions. SoTRAACE [22] aims at filling this gap providing an adaptable access control model encompassing both quantitative and qualitative risk evaluation.

### 2.2. SoTRAACE Risk Assessment and Access Control

**Figure 1:**
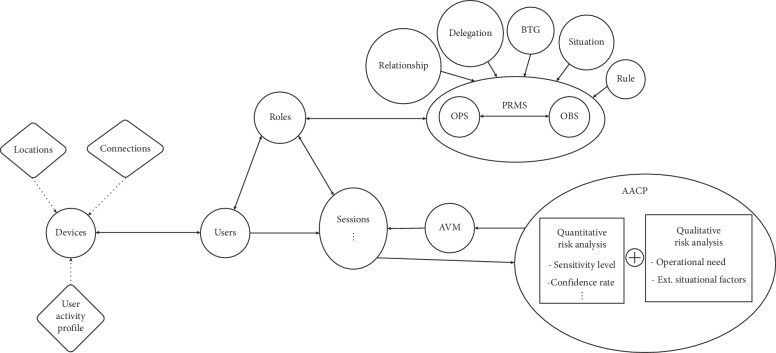
SoTRAACE architecture and components [22].

Figure 1 presents the generic architecture of the Socio-Technical Risk-Adaptable Access Control (SoTRAACE) model [22] based on RBAC.

New components that distinguish this model from the other access control models include devices, user activity profile (UAP), locations, connections, Adaptable Visualization Module (AVM) [31], and Adaptable Access Control Policy (AACP). Succinctly, SoTRAACE aims to automatically learn from individuals' interactions and from live data collected from every interaction a user makes comprising human, social, and technical context at that moment (e.g., time, location, previous interactions, and type of connection/device) and decides what is the most transparent, secure, and usable way (AVM) to both ask and retrieve the results of each request, to and from the application at hand. SoTRAACE performs a quantitative and qualitative risk assessment analysis supporting decision-making (AACP) on the most secure, private, and usable way to access and display information. More details about these components and their integration are available in previous research [22].

There are two types of risk assessment: quantitative and qualitative. The former uses numbers to quantify mostly the loss of tangible assets (e.g., replace a defective server) while the latter assesses the probability that a certain level of loss of confidentiality, integrity, or availability (e.g., low, moderate, and high) may occur and the impact it can cause (e.g., patient records are breached via hacking) [4]. Besides the international standards on risk management and risk assessment [4, 32], some examples of specific risk assessment frameworks for access control include (a) fuzzy multi-level security (MLS) access control model [33] that quantifies the risk associated with an access with basis on a value of information and probability of unauthorized disclosure, (b) a framework for threat assessment approaches for subject-object accesses, which can be selected based on the context of applications or on the preference of organizations [34], (c) RAdAC [29] (already introduced), or (d) DREAD (damage potential, reproducibility, exploitability, affected users, and discoverability) [35], which rates risk by answering five questions related with those five categories. The last two methods are reused by SoTRAACE and included in its own risk assessment mechanism.

## 3. Methods

A comprehensive search on subjects such as access control and risk evaluation (both quantitative and qualitative), specifically applied to mobile Apps, was performed so as to understand what types of risk assessment are used and how these can be improved (more details in Sections 1 and 2).

Since that search emphasized a lack of definition of security data categorization, especially in the heterogeneous domain of healthcare, a Delphi study was performed to allow the first definition of such categorization to be used within the mHealth SoTRAACE prototype in terms of quantitative risk. The Delphi method [36] is a structured communication method, which relies on a panel of experts in the specific research domain to answer a questionnaire in a structured, systematic, iterative, and anonymous way.

For this work, the Delphi study comprised a total of twelve (12) experts. The group comprised 3 (25%) females and 9 (75%) males with a background in computer science (*n* = 11; 92%) and electrical engineering (*n* = 1; 8%), with the expertise in computer and information security (*n* = 8; 66%), cryptography (*n* = 1; 8.5%), standards and modelling (*n* = 1; 8.5%), software development (*n* = 1; 8.5%), and information systems and computer engineering (*n* = 1; 8.5%). Half of the experts had experience between five to ten years (*n* = 6), while four of them had less than five years' experience and two had more than ten years' experience, in their fields of research/work. More experts work in academy/education (*n* = 9) than in the industry (*n* = 3). The expert group provided health data security categorization for a number of patient-related data, as well as the definition of the impact of those data security and privacy loss.

The questionnaire comprised twelve questions, eleven of which use a five-point Likert scale, from negligible (1) to critical (5), and the last question was an open question that allowed the experts to provide or suggest more information regarding evaluated factors or others that were not included in the study. That questionnaire was answered twice in two-time separated rounds, by the same group of experts, and at the end of the first round, an anonymized summary of the experts' results was provided to them. With the provided summary, the experts were encouraged to revise their earlier answers in the light of other members' replies so that, during this process, the range of the answers will decrease and the group will converge towards the most consensual answer. This type of study can help bringing the knowledge of security experts closer to real-life situations and their associated risks. The applied questionnaire and detailed rounds of questions are presented in the Annex and its results are presented in Section 4.1.1.

With the definition of the quantitative and qualitative risk assessment feature, a set of patient-access use cases was defined to implement and validate the proof of concept of the SoTRAACE prototype.

## 4. Results

In this section, we present results from the architecture, requirements, and implementation of risk assessment into the SoTRAACE prototype, as well as the description of patient use cases and how this can reflect into an mHealth application.

### 4.1. SoTRAACE and Risk Assessment

For the SoTRAACE model, risk assessment features were included within the Adaptable Access Control Policy (AACP) component, see Figure 1. Currently, SoTRAACE integrates a base definition for the core characteristics of security risk evaluation, operational need, external situation factors, and adaptable access control decisions from [29]. To quantify the security risk of each request, the AACP aggregates, in real time, all attributes that are instantiated in the session, namely, connection, location, and the UAP (user activity profile) from the device. It can also aggregate descriptive metadata from the object when available (e.g., type, sensitivity level of the requested resource, owner, and institution/company related), as well as the object logs (who/when/where that object was accessed or changed). Each attribute used to quantify the risk can contain exploitable threats. A very simple quantitative risk analysis could be used to calculate an average weight of the attributes gathered by the model. Nevertheless, more complex risk assessments can also be adopted according to data classification and their degree of sensitivity.

#### 4.1.1. Delphi Study for mHealth

**Figure 2:**
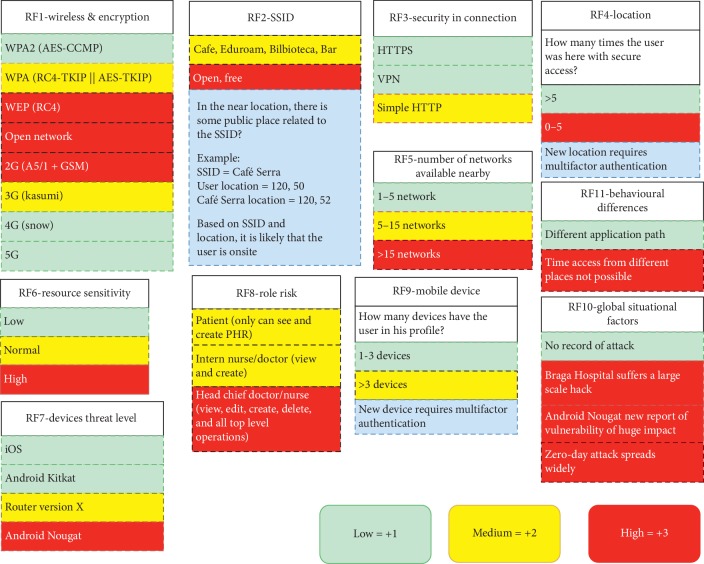
Risk factors and attributes analysed by the experts of the Delphi study (information with blue background comprises descriptive text).

The Delphi study performed in the scope of this work allowed the quantification of the relative importance of the risk associated with several technical, environmental, and contextual risk factors (RF). The selection of the eleven risk factors (Figure 2) was obtained from the following sources: (1) previous research work on the definition of SoTRAACE model (Figure 1) with new components (e.g., devices, locations, and connections) that integrate specific requirements, which can be associated with specific vulnerabilities (e.g., RF1, RF2, RF3, RF4, and RF7), (2) RFC documents on cryptographic and authentication protocols (https://www.ietf.org/rfc/rfc1994.txt and https://www.ietf.org/rfc/rfc5246.txt) (e.g., RF1 and RF3), and (3) debates among research experts in the domains of healthcare and information system security, during the course of the work, where attributes closer to the healthcare domain—RF6 and RF8—and human interaction—RF5, RF9, RF10, and RF11—were also defined. Some of the risk factors reflect vulnerabilities associated to Android mobile operating system because the actual Android market share is roughly in the neighbourhood of 75 to 85 percent, and it is expected that the gap towards IoS keeps increasing in the next years. However, similar attributes for other technologies, or types of devices, can also be adopted to calculate the necessary risk in different platforms and with different characteristics.

Each question answered by the security experts in the Delphi study was related with each of those RFs, which are RF1 (wireless and encryption), RF2 (SSID), RF3 (security in connection), RF4 (location), RF5 (number of networks available nearby), RF6 (resource sensitivity), RF7 (device threat level), RF8 (role), RF9 (registered mobile devices), RF10 (global situational factors), and RF11 (behavioral differences). Each RF comprises several attributes for the security experts to weight in terms of their risk criticality (e.g., attributes for RF3 are VPN, HTTPS, or HTTP), see Figure 2.

**Figure 3:**
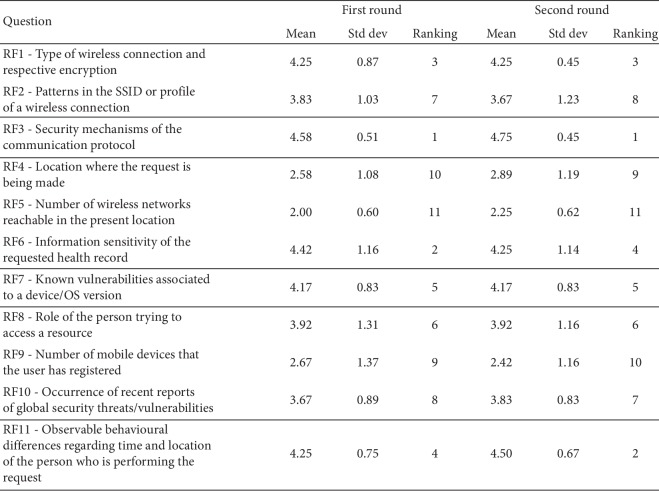
Delphi study: experts' review scores of the eleven risk factors for the two rounds.

The results, including means and standard deviations of each question of the two rounds, are presented in Figure 3. As expected, the standard deviation in most cases was reduced from the first to the second round, converging to a consensus of opinion among the experts. The risk was ranked using a number and color scale of low = 1 (green), medium = 2 (yellow), and high = 3 (red). This ranking was chosen by the authors as the simplest example scale for the purpose of using them as input in the SoTRAACE model to test its risk assessment features.

The respective final results extracted from the Delphi final round are converted to weights and are reallocated in the following arithmetic mean to calculate the risk:

(1) $$\begin{array}{c} \text{Risk}=\frac{\sum_{i=1}^{11}{\text{RF}}_{i}\times W_{i}}{\sum_{i=1}^{11}W_{i}}, \end{array}$$

where RF_*i*_ is the risk ranking (e.g., low = 1, medium = 2, or high = 3) associated to the respective risk factor *i*, in Figure 2, and *W*_*i*_ is the weight agreed by the experts in the Delphi study, for RF_*i*_. For instance, if the RF_1_-identified attribute is WEP (RC4) (Figure 2), its ranking would be high; so, for the purpose of calculating the risk, RF_1_ = 3, while the corresponding *W*_1_ = 4.25 (Figure 3).

The weighted mean ensures a dynamic variation of the number of risk factor collected and can never be null as some of the risk factors are always applicable, such as the SSID, type of wireless connection, data sensitivity, or type of device. These factors can be collected by the application, independently of the user.

Finally, the risk level output is a number between 1 and 3 and can be mapped to new security measures and access restrictions as in the following examples:

1. In all requests, give feedback to the user about the most unsecure attribute in the request (e.g., send message advising that Wi-Fi Protected Access (WPA) 2 is more secure than an open public Wi-Fi network).
2. If risk ≤ 1.6, usually no restrictions will be applied.
3. If risk > 1.6 and risk ≤ 2.2, the system will use authenticated encryption with associated data (AEAD) [37] to guarantee end to end encryption, regardless of other encryption mechanisms that may be in place by the network infrastructure.
4. If risk > 2.2, the system will use AEAD and the access to resources with high sensitivity will be denied.
5. BTG requests: besides having extreme risk, the urgent situation of the request always requires access to the data. These requests always use AEAD, and the data are fragmented and sent in different parts. At the client side, the various parts are merged and the complete data are presented.

More levels of security/sensitivity could be added, if necessary, to add more or less fine-grained possibilities. The cut values of 1.6 and 2.2 are just examples; they can be adjusted according to data sensitivity and the weight that needs to be put into each of the values of the scale (low, medium, and high).

**Figure 4:**
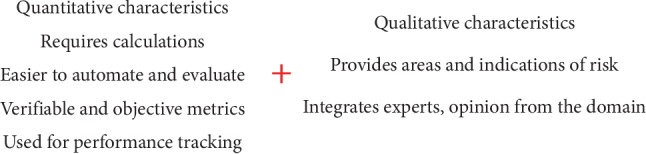
Hybrid risk assessment calculation using the results from the Delphi study (extract from [21]).

Following quantitative and qualitative calculations of risk, as defined by standards such as ISO/IEC 27005:2011 [32] and NIST SP800-30 Rev.1 [4], the Delphi study results provide, this way, means to use a hybrid risk assessment method, which combines characteristics from both quantitative and qualitative risk assessments (Figure 4). This helps integrating benefits from both worlds, thus leading to the adoption of more accurate and prioritized mitigation measures for the analysed domain. This can also be helpful in order to perform stronger statements to the organization's management and governance departments, as numbers sometimes can be clearer than descriptions and adjectives. Moreover, this still leaves space for stricter qualitative assessments, which may, for instance, include situations where specific values cannot easily be associated to risks (please see Section 4.1.2).

#### 4.1.2. Qualitative Risk Assessment and Access Decisions

Quantitative risk analysis is complemented with more qualitative measures that can integrate the operational urgency (how urgent is it to access that object at that moment?) and other external situational factors (unusual distant locations where the access was performed) to provide a more accurate, secure, and adapted access decision. For instance, a scenario illustrating a qualitative risk evaluation could be the following: if a nurse is trying to access a medical record at a different time from her normal working hours, using a different device and connection, the calculated quantitative risk will be higher than usual. However, a more qualitative analysis may attenuate that risk if it confirms that the nurse is accessing data that are customary and from a secure location (e.g., secure service provider Wi-Fi at home). In this case, auditing can register some warnings and visual security restrictions can be applied as a preventive measure.

After having assessed the risk, AACP specifies a set of rules (the decision) that can be applied to the permissions module (PRMS) for future reuse, if necessary. Decisions can vary according to the type of user, security and privacy requirements, and type of device or data sensitivity. Some examples of SoTRAACE decisions can be as follows: (1) simply block or allow the access (traditional access control), (2) enforce the fragmentation of the requested object and just allow access to some fragments (security visualization with adaptable visualization module (AVM)), (3) block or allow one or more operations to the object, or (4) trigger other hidden security protocols to better avoid the risk without compromising availability.

Finally, past decisions and respective parameters provided by the AACP are recorded and used to help decide each subsequent decision. The main goal is to analyse user-profiling information to securely improve and optimize similar future interactions. This knowledge can enhance algorithms that determine the risk, operational need, and the rate of positive/negative access control decisions, to build more accurate user activity profiles (UAP) and object logs and also, therefore, improve and monitor security measures in place.

### 4.2. SoTRAACE Prototype

#### 4.2.1. Architecture and Security Requirements

**Figure 5:**
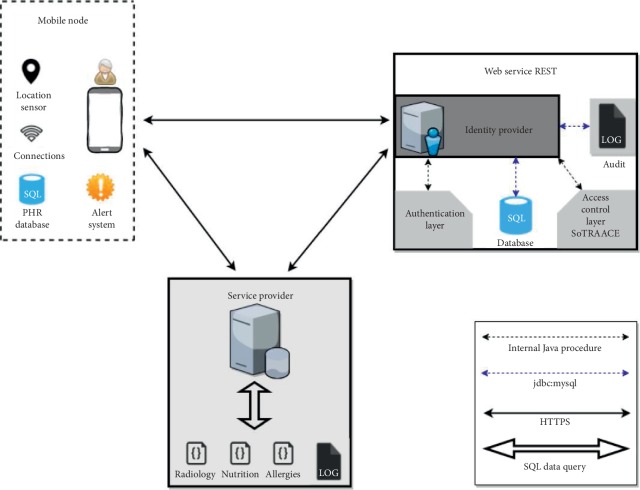
SoTRAACE prototype generic architecture.

The developed system is divided in three major components: mobile applications, web services (including identity providers, IdPs), and service providers (SPs) with databases. The patient mobile component comes with an application, with web-based central authentication and authorization IdP, to secure access and share health data stored in databases of geographically fragmented SPs. In Figure 5, the generic architecture is graphically schematized, with the respective representation of the different types of communications that are used.

The proof of concept was implemented on the operating system Ubuntu 16.04 LTS, and for the mobile application, Android was used. The native programming language used in Android development is Java. The integrated development environment (IDE) used was Android Studio, the official IDE for Google's Android OS development. The local data records on the mobile device were stored under SQLite database, and the Android layouts were designed using XML. To handle the asynchronous Android client requests, the Android application uses loopJ Android Asynchronous HTTP Client library. This provides an asynchronous call back-based HTTP and HTTPS client for Android built on top of Apache HttpClient libraries. The mobile apps were tested in the Android versions Marshmallow and Nougat and with a physical device Huawei p9 lite.

For the Web service, the technology used was RESTful API, which is a flexible way to provide different kinds of applications with data formatted in a standardized way and very important for eHealth, as it helps to meet integration requirements that are critical to building systems where data can be quickly combined and extended. Also, it can facilitate its use as it provides JSON format. IDE Eclipse Neon Enterprise Edition was used to build the Java Web service and configured to use Apache Tomcat servlet container (often referred to as Tomcat server). In addition, the Web service uses Jersey libraries and tools. Jersey RESTful Web service framework is open source, made in Java, which provides support for JAX-RS APIs and serves as a JAX-RS (JSR311 and JSR 339) reference implementation.

For the repository, a relational database management system (RDBMS) MySQL was used together with the phpMyAdmin administration tools. To connect the Java-based Web services (IdP and SPs) to the MySQL database, the official driver connector Java Database Connectivity (JDBC) was used. To test the RESTful Web services (IdP and SPs), Advanced REST Client was used as it makes a connection directly to the socket giving full control over the connection and URL request/response headers. This way, it is possible to analyze and test all headers before inserting them in the mobile applications.

For the risk-adaptable decisions in the access control layer, the Android application needs to manage data about users' locations and connections. Also, location is used in the authentication layer to identify the user. The alert system warns the user about some important aspects of the interactions. It can be used to (a) warn about the risk of access to specific data in a dangerous context, (b) release alerts to teach the patient to get better decisions and security, (c) warn about the existence of a new PHR (or EHR, electronic health record) or a change in one, (d) inform the user of new access requests, and so on.

Each IdP contains an authentication layer (to manage authentication of users and control their identity), an access control layer (based on SoTRAACE), and an SQL database to store the access-control list (ACL) permissions and user profile (with all past requests and attributes), as well as to assist the layers of authentication and access control. It also integrates a log system to enable audit.

SPs use SQL databases to store health data and logs. Those databases also store ACL permissions that are synchronized with the main service, the IdP. In each SP, the respective logs for posterior auditing are also stored. It is important to store information to a better version of control over the health data (e.g., who, when, and where the data was changed).

The ACL exists in the IdP, but it must also exist in the federated SPs. For instance, when the Internet connection fails in an institution, the internal Local Area Network (LAN) of the institution checks the internal ACL database for permissions, without the need to connect to an outside IdP. The database in the IdP is the one that contains the main ACLs. The health data are stored in institutional databases. The local SP ACL is synchronized with the main ACL in the IdP. For the system and services to remain available to authorized parties, a set of IdPs must exist. If one fails, another takes its place. The system can have many geographically fragmented federated institutions (SPs) that can share data between them, if the user consents. The user should be obligated to perform a login each time he/she requires a PHR from a different institution. As such, by using SSO, the users can move between services securely and uninterruptedly without specifying their credentials every time. Also, multifactor authentication must be present in the system to protect stolen devices and access from new locations in cases of stolen accounts.

The patient should have access to all clinical documents, history, and logs. Questions such as who, when, and where his/her documents were accessed and who changed them must always be recorded and available to the patients. There are some cases in which one cannot waste time setting permissions on the mobile phone. In cases of extreme emergency, such as an unconscious patient in an ambulance unable to give access to resources, the BTG mechanism needs to allow emergency access to the health professionals. This BTG access must be well defined and always recorded in the logs. Secure backups must also be performed with short intervals of time.

The management of health data can be critical, so all the end points and communications must be protected. Security mechanisms must assure confidentiality, integrity, and availability of patients' personal and health data, empowering this way patients' privacy. Besides patient data, attributes such as location, types of devices, and sensitive profile information must also be protected. To assure this, the AACP is embedded at the IdPs and all attributes are aggregated there, never reaching the SPs. These attributes help SoTRAACE to perform a risk evaluation and perform the best access decision at the moment of each request.

To develop the SoTRAACE prototype into a mobile application, an analysis of global smartphone OS market was performed and it was decided that the initial OS target for the implementation and test of this research is Android [38].

#### 4.2.2. Patient Use Cases and Proof of Concept

**Figure 6:**
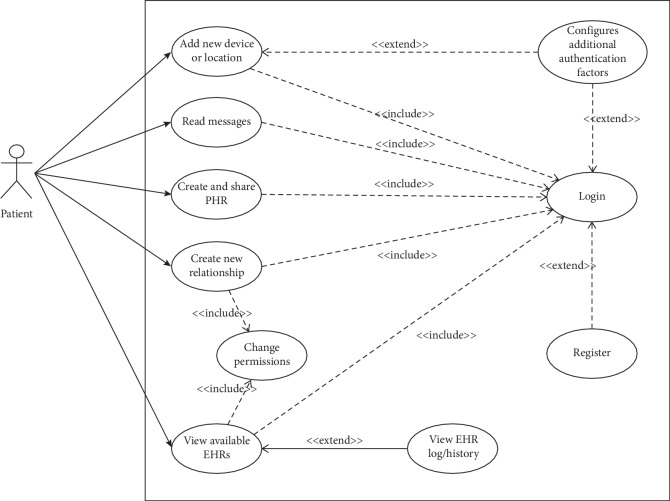
Overview of the various actions the patient can perform with the SoTRAACE prototype.

We resort to use case diagrams to provide an overview on how a user interacts with the features of the system and the functionality provided by the system in terms of actors. A patient, with the SoTRAACE prototype application installed in the Android mobile device, can try the various options described in Figure 6. For instance, the login action includes a previously registered account, and if the location and device IMEI are new, the login can be extended to include a multifactor authentication protocol.

**Figure 7:**
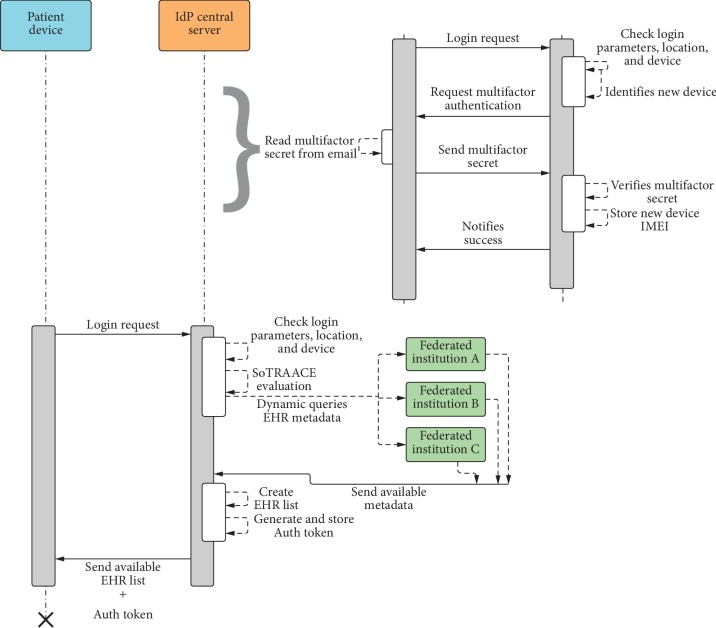
Patient login sequence diagram and multifactor authentication part (bracket), when required.

The UML sequence diagram in Figure 7 shows the sequence of a patient's login functionality to access the mobile application. As a prerequirement, the patient needs to have a registered account. In the first step, the patient requests to login, sending his/her login credentials, also the mobile application collects the GPS location (if available) and device IMEI (to identify the device). Next, the IdP validates the login credentials and checks the patient's profile to see whether the device and location are already known or were previously used. If not, multifactor authentication will be used instead (Figure 7, additional bracket part on the right). After the login stage, the patient can choose between the options: (i) add new device or location, (ii) read messages, (iii) create PHR, (iv) create new relationships, and (v) view available PHRs/EHRs.

For the multifactor authentication, the patient receives a random secret PIN in his/her email; each time, the multifactor authentication is required. When the patient requests login, the IdP verifies if he/she is using a new, nonregistered device or location (i.e., not registered in their profile, from past accesses) and requests a multifactor authentication to the patient. The patient checks his/her email and sends the secret PIN to the IdP, which verifies this secret authenticity. If everything matches, IdP stores the new device IMEI or/and location in the user profile and notifies the patient. Now, the patient can login using a different mobile device.

Since a main objective of this prototype system is to ensure patients' privacy and empowerment, SoTRAACE collects available user data request/interaction (e.g., location and connection) to perform risk evaluation and agree on the most adequate access decision. However, to assure privacy, personal data cannot be stored on the federated institutions' (SP) side but must always be stored at the IdP. Moreover, each request generates a log to help build the patient profile, the user activity profile (UAP), and to enable audit.

**Figure 8:**
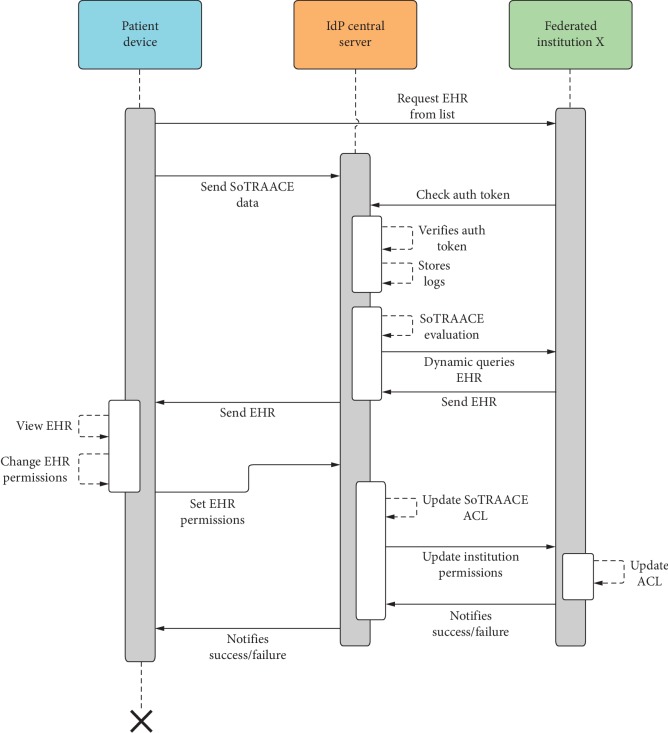
Patient EHR access with permission change sequence diagram.

Figure 8 shows a sequence concerning a patient choosing an EHR from the initial menu list. This request goes with an authorization token (AT) directly to the federated institution. The patient's device sends the necessary data to the IdP for SoTRAACE to do the work. The federated institution validates the AT with the IdP. If the AT is valid, a record is created in the log system. After this, SoTRAACE evaluates the risk for that request and adopts the query (e.g., if the risk is high, some parts of the EHR are omitted). Then, the EHR is sent to the IdP, and the IdP determines the best protection and accesses decision based on SoTRAACE and sends it to the patient. The patient views the EHR and changes the access permissions. Those permission changes are updated in the IdP SoTRAACE ACL and at the federated institution ACL. Finally, the patient is notified about the success/failure of his/her alterations.

**Figure 9:**
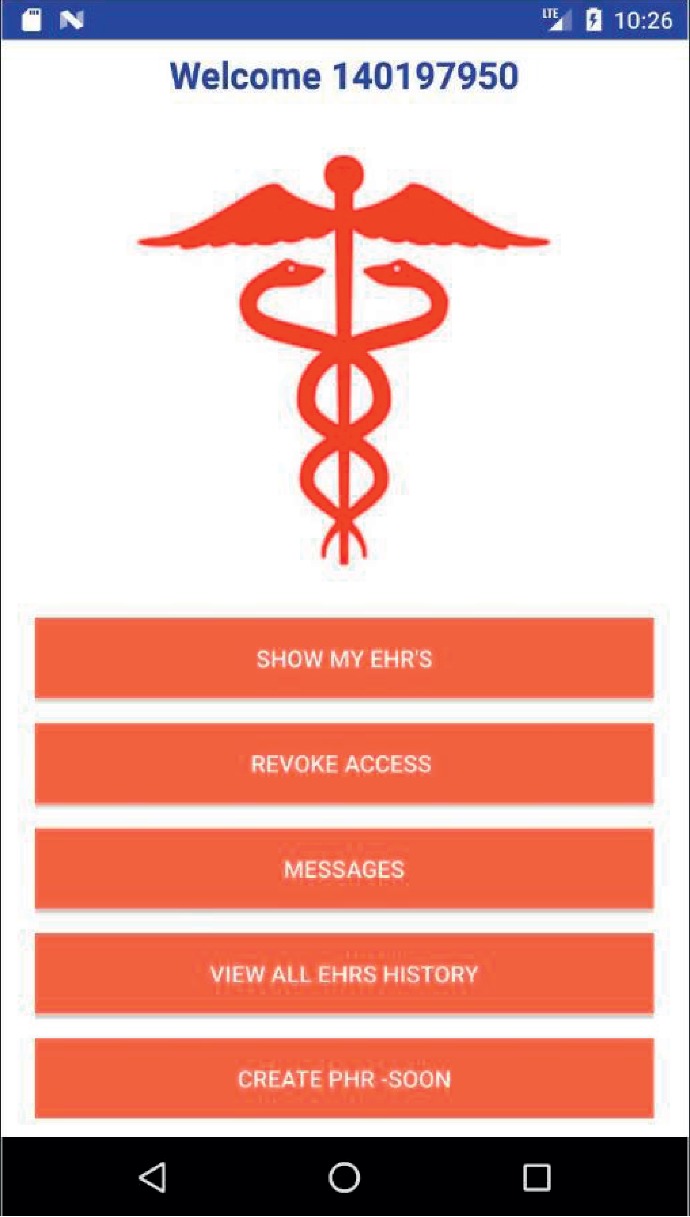
Mobile application main menu for the patient.

**Figure 10:**
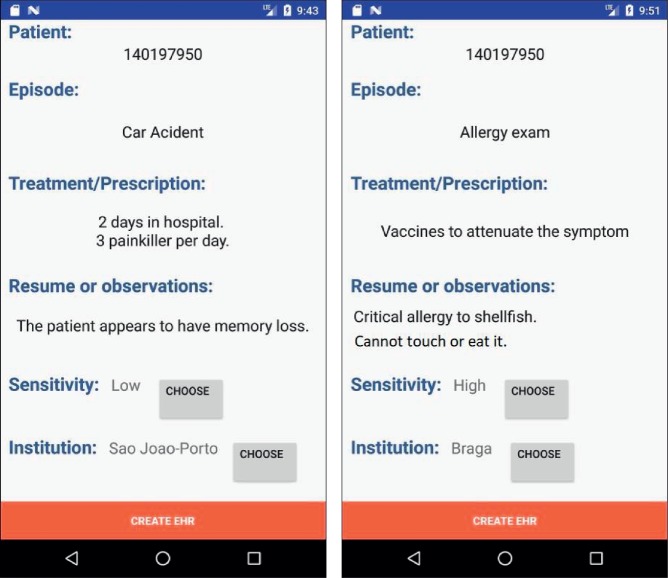
Patients can view different parts of their EHRs and their associated level of risk.

Figure 9 shows a screenshot of the main page of the patient's mobile SoTRAACE prototype with the main menu activity and available functionalities.  Figure 10 shows the displays that a patient sees when the functionality “SHOW MY EHR's” is selected. On the left is an EHR for the internment of the patient within a hospital and related actions. There is the possibility to verify the type of sensitivity level associated to this record. The same is true for the image on the right, which shows similar data but for allergy exams.

## 5. Discussion

On the subject of health data, associated risks are commonly focused on people's diagnosis, treatments' outcomes, and to help in medical decisions. However, risks need to be associated and constantly evaluated in relation to additional health data that are accessed and processed by any means (e.g., paper, Web, and mobile) as well as for each type of interaction and associated contextual variables/characteristics. This assumes a higher criticality due to the fact that those data can also greatly influence people's health, security, and privacy. To achieve this, risk assessment should always be considered, which is not happening at the moment. To make matters worse, although the use of mHealth can improve treatment and outcomes and change the paradigm of healthcare to anytime/anywhere, it can also exponentially increase the available vulnerabilities and threats and, again, the risk.

With all this in mind, the authors propose a more adaptable/adequate means for risk assessment on the fly integrated into existing access control models but with novel functionalities to perform a more complete risk assessment for each mHealth interaction.

The authors could not find such models for other applications, but certainly not for mHealth, which could integrate all the necessary requirements both for flexibility and adaptability, as well as qualitative and quantitative risk analysis, specifically for personal health data. Furthermore, no guidelines or standards for this specific domain were found to help define data sensitivity for mobile healthcare use and associated visualization/presentation.

This work presents SoTRAACE, an access control decision model which integrates more features for flexibility and better adaptable security, not only for calculating hybrid risk assessment for each users' contextual interaction and subsequent communications but also to improve and adapt visualization with end users. SoTRAACE, associated with the Delphi study presented in this work, provides a first effort to achieve the categorization of personal health data resorting to security experts to both reflect that experts' knowledge and to be closer to real-life situations and their associated risks.

It is clear that integrating a series of security and sensitivity level classification to a decision process is not difficult; the difficulty lies on the definition of those levels for each type of data and how accurate and adaptable they can be. To do this, it is important to compare and discuss results from other works. In this case, we found a work where some similarities can be drawn, which also helps validating our own results. In work [39], interviews were applied to sixteen participants, the majority having expertise in ICT, where questions focused on the threats, criticality, and frequency of those threats, in healthcare information systems. Although the questions were generic and were not rated in several rounds, with the aim to provide risk calculation, as in our Delphi study, the main outcome was a list of the most common identified threats, by those participants. The obtained results are closely associated to contextual and environmental attributes as well as to the specific technologies that are used to assess those threats, as most critical identified threats for that study comprise power, Internet, and air conditioning failures. Nonetheless, the most five critical threats identified in that study overlap in some degree with some of the most significant risk factors ranked by our experts. These overlaps are highlighted in Table 1.

Finally, the performed Delphi study shows that much more work needs to be done. There is the need for the whole community of security and healthcare experts to join in the definition of security/sensitivity levels of relevant variables for the decision process. In this case, it was only an initial effort and the experts could only focus on very specific (mostly technology related) aspects of security (e.g., wireless connections, communication protocols, and users' roles).

This is where the main research efforts need to be focused. Once this is clearly defined, its consensual implementation in the clinical practice will not take major resources, as technology is readily available to model the identified needs. The presented prototype corroborates the previous statement since it was implemented with existing technology and security protocols. Moreover, if given an initial strong base to perform adaptable decisions, the access control model will then learn and optimize with its use time and provide personal and customized secure access control for all mHealth users.

### 5.1. Limitations

One limitation of this work concerns the lack of existing models or hybrid risk assessment procedures and related categorized data in terms of security and sensitivity levels in healthcare, to perform better and more adapted access control decisions. There are no means to compare with proposed work, and therefore, this constitutes an initial step in that direction. Being such a first attempt, this work does not yet integrate the prototype testing with real users and in real scenarios, though this is planned, as future work, once the prototype is enriched with all the required use cases.

Another limitation is the fact that the Delphi study was performed only with security experts and did not integrate multidisciplinary expertise, such as the one from healthcare professionals. However, this expertise was integrated within this study, when defining what risk factors to evaluate. Another limitation was the number of analysed risk factors, which was small and needs to include more contextual as well as clinical and health-related data aspects.

The presented prototype screenshots are very simple. At this stage, there was no need to show more complex application functionalities as the main goal of this work was focused on risk assessment procedures for mHealth applications.

## 6. Conclusions

Building adaptable and resilient access control models into the most generalized technology used nowadays (e.g., smartphones) is crucial to fulfil both users' goals as well as security and privacy requirements for healthcare data. This work is an alert for the research community to put more efforts into these areas in order to better integrate and personalize security into every patient or, for that matter, any type of user's life.

## Figures and Tables

**Table 1:** Comparison of the most critical identified risks by the Delphi study and the most common threats in healthcare information systems, identified by Samy et al. [39].

Delphi study risks	Threats from Samy et al. [39]	Overlapping
(1) Security in the communication	(1) Power failure/loss	Availability issues of the communication channel
(2) Behavioural differences (4) Resource sensitivity	(2) Acts of human error or failure	Human behaviour affecting integrity and security of medical records
(5) Device threat level	(3) Technological obsolescence (4) Hardware failures or errors (5) Software failures or errors	Software and hardware used in the devices can comprise unpatched security vulnerabilities

## Data Availability

The resulting data from the Delphi method, used to support the findings of this study, are included within the article. The survey to collect data for the Delphi method used to support the findings of this study is provided in Supplementary Materials ([Supplementary-material supplementary-material-1]).

## Supplementary Materials

Supplementary Materials This includes survey security risk.
