# Supplementary material for: Assessing Access Control Risk for mHealth: A Delphi Study to Categorize Security of Health Data and Provide Risk Assessment for Mobile Apps
Source: J Healthc Eng. 2020 Jan 16;2020:5601068. doi: 10.1155/2020/5601068 (PMC6988678; doi:10.1155/2020/5601068)
Supplement: Supplementary Materials — This includes survey security risk. [file 5601068.f1.pdf]

ANNEX

Security risk evaluation in Health Information Systems

This survey is part of a Delphi study developed in the context of a MSc dissertation in computer science and engineering. Its purpose is to identify and classify risk factors in the access to electronic health records. The objective is to obtain a deeper insight about the effective and perceived risk associated with some of the environmental variables of each request.

Please classify the impact that the referred attribute can have in the overall security of a health information system.

*Required

1. **Type of wireless connection and respective encryption (e.g. WEP with RC4, WPA2 with AES, 3G with Kasumi).** *
   Mark only one oval.

	1	2	3	4	5	
Negligible	◯	◯	◯	◯	◯	Critical

2. **Patterns in the SSID or profile of a wireless connection (e.g. free, open, coffee, guest, hotspot, home, public).** *
   Mark only one oval.

	1	2	3	4	5	
Negligible	◯	◯	◯	◯	◯	Critical

3. **Security mechanisms of the communication protocol (e.g. HTTP, HTTPS, connection via VPNs).** *
   Mark only one oval.

	1	2	3	4	5	
Negligible	◯	◯	◯	◯	◯	Critical

4. **Location where the request is being made (e.g. big cities, public places, schools).** *
   Mark only one oval.

	1	2	3	4	5	
Negligible	◯	◯	◯	◯	◯	Critical

5. **Number of wireless networks reachable in the present location.** *
   Mark only one oval.

	1	2	3	4	5	
Negligible	◯	◯	◯	◯	◯	Critical

6. **Information sensitivity of the requested health record (e.g. public, personal or private data).** *
   Mark only one oval.

	1	2	3	4	5	
Negligible	◯	◯	◯	◯	◯	Critical

7. **Known vulnerabilities associated to a device / OS version (e.g. IOS, Android nougat or marshmallow).** *
   Mark only one oval.

	1	2	3	4	5	
Negligible	◯	◯	◯	◯	◯	Critical

8. **Role of the person trying to access a resource (e.g. physician, nurse, chiropractor, operator of ambulance, chief doctor, patient).** *
Mark only one oval.

	1	2	3	4	5	
Negligible	⬭	⬭	⬭	⬭	⬭	Critical

9. **Number of mobile devices that the user has registered.** *
Mark only one oval.

	1	2	3	4	5	
Negligible	⬭	⬭	⬭	⬭	⬭	Critical

10. **Occurrence of recent reports of global security threats and vulnerabilities.** *
Mark only one oval.

	1	2	3	4	5	
Negligible	⬭	⬭	⬭	⬭	⬭	Critical

11. **Observable behavioural differences regarding time and location of the person who is performing the request (e.g., login in Australia two hours after a successful request in Portugal).** *
Mark only one oval.

	1	2	3	4	5	
Negligible	⬭	⬭	⬭	⬭	⬭	Critical

12. **Comments or suggestions regarding these or other attributes that, in your opinion, are important for the risk evaluation.**
